# Novel Chemo-Photothermal Therapy in Breast Cancer Using Methotrexate-Loaded Folic Acid Conjugated Au@SiO_2_ Nanoparticles

**DOI:** 10.1186/s11671-020-3295-1

**Published:** 2020-03-19

**Authors:** Reza Agabeigi, Seyed Hossein Rasta, Mohammad Rahmati-Yamchi, Roya Salehi, Effat Alizadeh

**Affiliations:** 1 Department of Medical Biotechnology, Faculty of Advanced Medical Sciences, Tabriz University of Medical Sciences, Tabriz, Iran; 2 Department of Medical Bioengineering, Tabriz University of Medical Sciences, Tabriz, Iran; 3 Drug Applied Research Center and Department of Medical Nanotechnology, Faculty of Advanced Medical Sciences, Tabriz University of Medical Sciences, Tabriz, Iran

**Keywords:** Au@SiO_2_ nanoparticles, Low level laser therapy, Methotrexate, Folic acid, Breast cancer

## Abstract

Low level laser therapy (LLLT) is known as a safe type of phototherapy to target tumor tissue/cells. Besides, using targeted nanoparticles increases the successfulness of cancer therapy. This study was designed for investigating the combined effect of folate (FA)/Methotrexate (MTX) loaded silica coated gold (Au@SiO_2_) nanoparticles (NPs) and LLLT on the fight against breast cancer.

NPs were synthesized and characterized using FTIR, TEM and DLS-Zeta. The NPs had spherical morphology with mean diameter of around 25 nm and positive charge (+13.3 mV) while after conjugation with FA and MTX their net charge reduced to around -19.7 mV.

Our findings in cell uptake studies clearly showed enhanced cellular uptake of NPs after FA and MTX loaded NPs in both breast cancer cell lines especially on MDA-MB-231 due to high expression of folate receptors. The results indicated that LLLT had a proliferative effect on both breast cancer cell lines but in the presence of engineered breast cancer targeted nanoparticle, the efficacy of combination chemo-photothermal therapy was significantly increased using MTT assay (p<0.05), DAPI staining, and cell cycle findings. The highest apoptotic effect on breast cancer cell lines was observed in the cells exposed to a combination of MTX-FA loaded Au@SiO_2_ NP and LLLT proved by DAPI staining and cell cycle(by increasing the cell arrest in subG0/G1). Taken together a combination of chemotherapy and LLLT improves the potential of breast cancer therapy with minimum side effects.

## Introduction

Breast cancer (BC), as the most frequent women, affecting cancer recently has reported with 1.7 million new cases worldwide [1]. Because of its complicated etiology and poor response to the treatment, often known to be the central cause of cancer associated deaths of women universally [2–5]. About 40,000 women in the U.S. were predictable to die from BC in 2014 [2, 6, 7] “(www.cancer.org)”. With the 522,000 deaths overall, it is the fifth cause of death from cancer with around 800,000 cases in less developed and about the same frequency in developed regions [1]. In Asian countries the greatest age of beginning is among adults of 40 or 50 years in comparison with the western countries which is frequent among 60–70 years [8]. The major risk factors of BC are female sex, family history, age, and varying generative leanings, such as first childbirth at the age of more than 30 years, early menarche and later menopause, and nulliparity [9].

The main aim in the fight against cancer is developing effective therapeutic plans with low toxicities and high specificities to eliminate tumors, mainly their metastases, and further their recurrence prevention. But presently used cancer treatment approaches, such as surgery, chemotherapy and radiotherapy showed various side effects [10–12] and all fail to achieve this aim [13, 14]

The past few decades have observed major struggles in the treatment of cancer [15, 16]. Between the current popular therapeutic approaches, thermal therapy has grown as a prospective treatment method [17]. Recently photothermal therapy (PTT), as a potentially effective and non-invasive cancer therapy, has attracted significant attention [18, 19]. PTT based on photo-absorbing nanostructures has become a different way to the general methods [20, 21]. In a typical PTT, that use PTT agents to destroy tumor by getting enough hyperthermia (42°C) under laser irradiation (near-infrared (NIR) light in the range of 700–1100 nm), has been studied as a greatly precise and negligibly invasive method of cancer treatment [22–28].

A number of nanoparticles have been widely studied as imaging contrast agents, drug delivery carrier, and transformer of energy modalities such as laser, radio-waves, and ultrasound, to thermal phenomena responsible for therapeutic effects [29–39].

Gold nanoparticles have attracted great attention during the past decade due to their high localized surface plasmon resonance (LSPR) and easy surface conjugation with biomolecules [40]. They have revealed high-performance photothermal conversion capacity in the NIR area [41–43] without harmful side effects in biological systems [44].

Although gold nanoparticles have been recognized as a promising photo-synthesizer, but due to their poor photothermal stability upon repetitive NIR irradiation, gold nanoparticles gradually lose their photothermal converting ability that limited their use in clinical practice. In addition, gold nanoparticles are not good drug carriers either because of their poor drug loading capacity and controlled drug release profile [45, 46]. Alternatively, mesoporous silica nanoparticle (MSN) was known to be appropriate drug, DNA and protein carrier because of its higher drug loading capability and lack of toxic contents that produced from its degradations. They also have a big surface area, controllable size, highly available pore volume, and desired surface features apply for a modification [47].

Nanoparticles after conjugating to a chemotherapeutic agent and cancer targeting ligand can inhibit disadvantages of routine chemotherapy, such as un-specific delivery, poor water solubility, and low therapeutic indices [48, 49].

Agents showing chemotherapeutic properties such as doxorubicin, cyclophosphamide, methotrexate, fluorouracil, and docetaxel are used single-handedly or in combination as the main core treatments, or aided with other treatments like PTT. Most of cancer patients experiencing adverse effects of chemotherapy drugs due to their non-precise distribution in patient‘s body that affects all the organs. These drugs hurt some of the fast growing normal cells, for example blood cells, mucous membrane cells covering the internal organs, and hair follicles [50–53].

Methotrexate (MTX) has been the most frequently used drugs for rheumatoid arthritis and several types of tumors such as skin, lung, head and neck, and breast [54, 55]. It inhibits dihydrofolate reductase (DHFR), the enzyme contributing to the production of tetrahydrofolate and its byproducts which are necessary for thymidylate and purine synthesis and both of them are vital for cell growth and cell proliferation. Hence, blocking DHFR methotrexate prevents the synthesis of 4 basic macromolecules DNA, RNA, thymidylates, and proteins [56].

Unfortunately, alike to most conventional PTT agents, the main challenge is to attain selective accumulation of GNPs in the target tissue after systemic injection [57–59]. Targeted cancer therapy allows the delivery of chemotherapeutic drug to specific cancer cells while decreasing the exposure of normal healthy cells. This led us to deliver greater dosage of drug to cancer cells with lower systemic toxicity. Ligand targeted nanoparticles are precisely identified cancer cell markers, that are highly expressed on the cancer cell surface [40].

Folic acid (folate or vitamin B9) is a key material for cell growth and metabolism. Because of the great affinity of folate for the folate receptor proteins, it is utilized as an element for cancer targeting. Folate receptor, as a tumor biomarker, is overexpressed in definite malignant cells like breast, ovarian, lung, kidney, brain, and colon cancer [60]. Folate conjugated drug delivery systems enhance cellular uptake of drug via endocytosis [61].

## Materials and Method

### Reagents and materials

We used double distilled water (Ghazi Company, Tabriz, Iran) and analytical grade chemical reagents for our experiments. A number of reagents were purchased from Sigma-Aldrich Company including: Tetraethyl orthosilicate (TEOS, 98%), (3-Mercaptopropyl) trimethoxysilane (MPTES,95% purity), folic acid and rhodamine B. A group of materials was purchased from Merck Co: Hydrochloric Acid (HCl, 37%), Ammonia solution (25%), toluene Sodium hydroxide (NaOH, 98%), and further solvents. MTX was bought from Zahravi Farma Company, Tabriz, Iran.

### Instrumentation

In this study, for analyzing the particles size and morphology, transmission electron microscopy (TEM) (LEO 906, Germany) was employed. We prepared around 100μL of our nanoparticles suspended in aqueous solution at room temperatures. The solution was transferred onto a carbon film coated on a copper grid of TEM with subsequent freeze drying and observed at 80KV. Determination of particle size was performed by DLS (dynamic light scattering) measurement at 25 °C using a Zetasizer Nano ZS90, Malvern Instruments, Malvern, UK. The Zeta potential measurements for the prepared NPs were performed by photon correlation spectroscopy (Zetasizer-ZS, Malvern Instrument, UK). A double beam UV–Vis spectrophotometer (UV-1601 PC model SHIMADZU, Kyoto, Japan) was employed for absorbance measuring by 700μL quartz cuvette having 10 mm path length. Fourier transforms infrared (FTIR) spectroscopy from Bruker Tensor 27 spectrometer, Germany was utilized for KBr pellet method performing. The pH measurements were done with a Metrohm 713 pH-meter (Herisau, Switzerland). The mechanical stirrer Heidolph RZR 2102 Control Overhead Stirrer (Schwabach, Germany) was used for stirrings. The encapsulation efficiency of FA and MTX was calculated using the HPLC system consisted of Waters 2690 separation module equipped with a UV–vis detector Waters 2500 Pump 1000 detectors (Waters, Milford, MA). The chromatographic separation was implemented at ambient temperature using a C18 m Bondapak (250 mm 4.6 mm, 10 mm, 125 A Waters, Ireland) chromatography columns.

### Preparation of Au@SiO_2_ Nanoparticles

The SiO_2_ NPs were synthesized based on the sol–gel method reported previously [62, 63]. In the next step thiol-functionalized silica-coated nanoparticles (TFSNPs) were prepared according to the method mentioned in our previous study [64]. The Au nanoparticles were produced by a citrate reduction method (Turkevich method) [65]. Finally, the surface of TFSNPs was covered by AuNPs. At first TFSNPs were dispersed in water with the aid of the bath sonicator for at least one hour and added to the AuNPs solution and sonicated for additional 30 min. The reaction was done at dark condition for two days in dynamic stirring at 25 °C. The Au@SiO_2_ nanoparticles with purple colored appearance were collected by centrifugation (10000 rpm, 10 min) and dried in a vacuum oven.

### MTX and FA loading

MTX as well as FA were loaded into the Au@SiO_2_ nanocarrier as follows: MTX (10 mg) was added to a 10 mL well-dispersed suspension of nanocarrier in PBS (5 mg/mL, pH 7.4) and agitated moderately at room temperature for one day in dark conditions. The MTX loaded Au@SiO_2_ nanocarrier was collected by centrifugation. The supernatant was collected for the measurement of unloaded MTX. Then MTX loaded Au@SiO_2_ nanocarrier was dispersed in PBS (5 mg/ mL, pH 7.4) and FA (10mg) were added to the solution and stirred moderately at room temperature for another day in dark conditions. The FA-MTX loaded Au@SiO_2_ nanocarrier was gathered by centrifugation and the supernatant was separated for the calculation of unbound FA in the final step. The FA-MTX loaded Au@SiO_2_ nanocarrier was freeze-dried and stored for next experiments. The amounts of unbound MTX and FA were calculated using HPLC method by protocol reported previously [66]. Folic acid was dissolved in ammonium hydroxide (10 wt %) and diluted with the mobile phase. The retention time for MTX and Folic acid were 10.5 and 5.95 min, respectively. Triplicate samples were applied. The drug loading efficiency (DLE) was calculated by the following formulas:

1 $$ ee\left(\%\right)=\frac{\left( initial\ total\ drugs- Unabsorbed\ drugs\right)}{Initial\ total\ drugs}\times 100 $$

### Cell lines selection and culture

Two breast cancer cell lines of interest with reported surface expression levels folate receptor (FR) [67] including MCF-7 and MDA-MB-231 was selected and purchased from the Pasteur Cell Bank (Tehran, Iran) for the cytotoxicity investigations. The selected cell lines were grown in complete medium containing RPMI1640 (Thermoscientific), 10% Fetal bovine serum (FBS), and 1% Penstrep (Thermoscientific) in thermal and atmospheric conditions of 37°C, 5% CO_2_, and 95% humidity.

### Cell cytotoxicity assay

Cell viability assays were performed for measuring cell proliferation after different NPs treatments without laser irradiation. Briefly: the MCF-7 or MDA-MB-231 cells were plated in 96 microplates with cell density of 1.5×10^4^ for 24 h, then cells were treated with MTX, Au@SiO_2_ and FA-MTX loaded Au@SiO_2_ NPs. The cells without treatment were considered as control. At the next step, the tetrazolium dye MTT (Sigma) at final concentrations of 5 μg/ml was added to the cells and incubated at 37°C for 4 h. Then, the MTT solution was removed and the settled Furmazan crystals were dissolved in Dimethyl Sulfoxide (DMSO) (BioIdea, Iran) under gentle shaking for 10 min. Finally, the absorbance was measured in 570 nm by an ELISA reader. The viability of cells was normalized to control cells and background was removed by subtraction of blank measurements.

### In vitro laser therapy

For Low Level Laser Therapy (LLLT), 810 nm wavelength NIR laser (Diode laser Mustang 2000, Russia) with 185 mW output power dose was used for cancer cell destruction. At first MCF-7 and MDA-MB 231 cells with cell density of 1.5×10^4^ treated with the Au@SiO_2_ and FA-MTX loaded Au@SiO_2_ NPs then were exposed to laser irradiation with different laser doses (30, 60, 75, 90 and 105 J/cm^2^) and fixed exposure time (139 sec). The cells exposed to only laser irradiation (without NPs) and the cells without any NPs and laser treatments considered as positive and negative control, respectively. After 24 h of laser irradiation the cell viability was measured by MTT assay method [64].

### Nanoparticles cellular uptake assay

A detailed checking of the NPs cell internalization is essential in order to confirm the specific effect of surface modified nanocarrier for each cell line. In the present work, we employed both flow cytometry as quantitative and fluorescence microscopy for qualitative checking the up-take of NPs by MCF-7 and MDA-MB-231 cell lines.

For suspension of NPs, the rhodamine B (RhoD) solution in PBS was added along with stirring for 24 h at ambient temperature and dark room (preventing bleaching). Then, RhoD-loaded NPs were separated by Amicon Filter with nominal molecular weight limit (NMWL) of 30 kDa and centrifuged for 15 min at 5000 rpm and washed with PBS buffer to eliminate the unbounded RhoD. The cells were seeded in plates at the density of 5×10^5^ per well and let to reach confluence. The cells were treated with Rhodamin B-loaded NPs for 30, 90 and 180 minutes, non-treated cells used as control. Afterwards, the cells were trypsinized and washed with PBS, and then fluorescence was quantified with flow cytometric analysis (BD Biosciences FCASCalibur flow cytometer; BD Biosciences, San Jose, CA, USA). The Intracellular uptake of rhodamine B-labeled NP or NPD was further confirmed by fluorescence microscopy. MCF-7 and MDA-MB-231 cells were grown on coverslips and after 24 h cells were treated with free Au@SiO_2_ NPs and MTX-FA loaded Au@SiO_2_ NPs. After incubation for 30, 90 and 180 minutes, the cells were washed with PBS and rhodamine B labelled nanocarrier uptakes was observed using a fluorescence microscope (Olympus microscope Bh2- FCA, Japan).

### Apoptosis study by Fluorescence microscopy

One method for the nuclear qualitative study of apoptosis is a fluorescent dye DAPI which binds to DNA and is detectable by appropriate microscopic. We used a protocol as previously reported for DAPI staining [68], shortly: the MCF-7 or MDA-MB-231 cells were plated in 6 well format vessels at a density of 5 ×10^5^ and let them attach and grow for 24 hours. After treatment with MTX, Au@SiO_2_ NPs and MTX-FA loaded Au@SiO_2_ NPs with and without laser treatment the cells were washed with PBS (Sigma) and then subjected to fixation by 10% formaldehyde (Merck), next: cells were permeabilized with Triton X-100 (Sigma) for 15 minutes. After proper washings, the cells were stained with DAPI (sigma) for 5 min. Finally, the apoptotic nuclei (fragmented or wrinkled) were visualized by a Fluorescence microscope (Olympus). The cells without any treatment were considered as negative control and the cells received only laser irradiation as positive control.

### Investigations of cell cycle disturbances

The MCF-7 and MDA-MB-231 cell cycle distributions were determined by flowcytometry analysis. In this way, the cells were seeded with starting populations of 5 × 10^5^ and allowed to reach 80% confluence. Subsequently, the cells treated with MTX, Au@SiO_2_ NPs and MTX-FA loaded Au@SiO_2_ NPs with and without laser irradiation was performed. The cells without any treatment were considered as negative control and the cells received only laser irradiation as positive control. Then, the cells were harvested by trypsinization following with proper PBS washings. Next, the cells were fixed by Ethanol (Merck) for 48 hours. At the next step, the fixed cells were washed, then treated with Ribonuclease A (Cinaclone), with subsequent addition of propidium iodide (PI) (Sigma) at dark. The fluorescence signals were detected by FACS set from Beckton Dicinson Company.

### Statistics of the study

The experiments for each step have been performed in three repeats and the results were reported as mean ±SD. The ANOVA was used for comparison of significance among groups. The differences were reflected significances where probability value was calculated <0.05 by SPSS software.

## Results and discussion

### Characterization of synthesized NPs

**Fig. 1:**
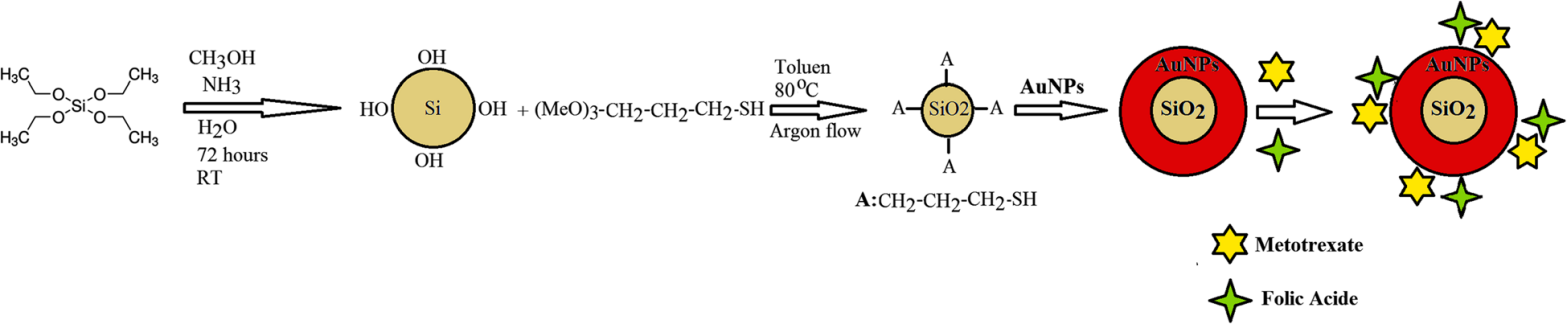
The stepwise synthetic scheme for the preparation of folate and methotrexate loaded biocompatible Au@SiO_2_ NPs nanoparticles

**Fig. 2:**
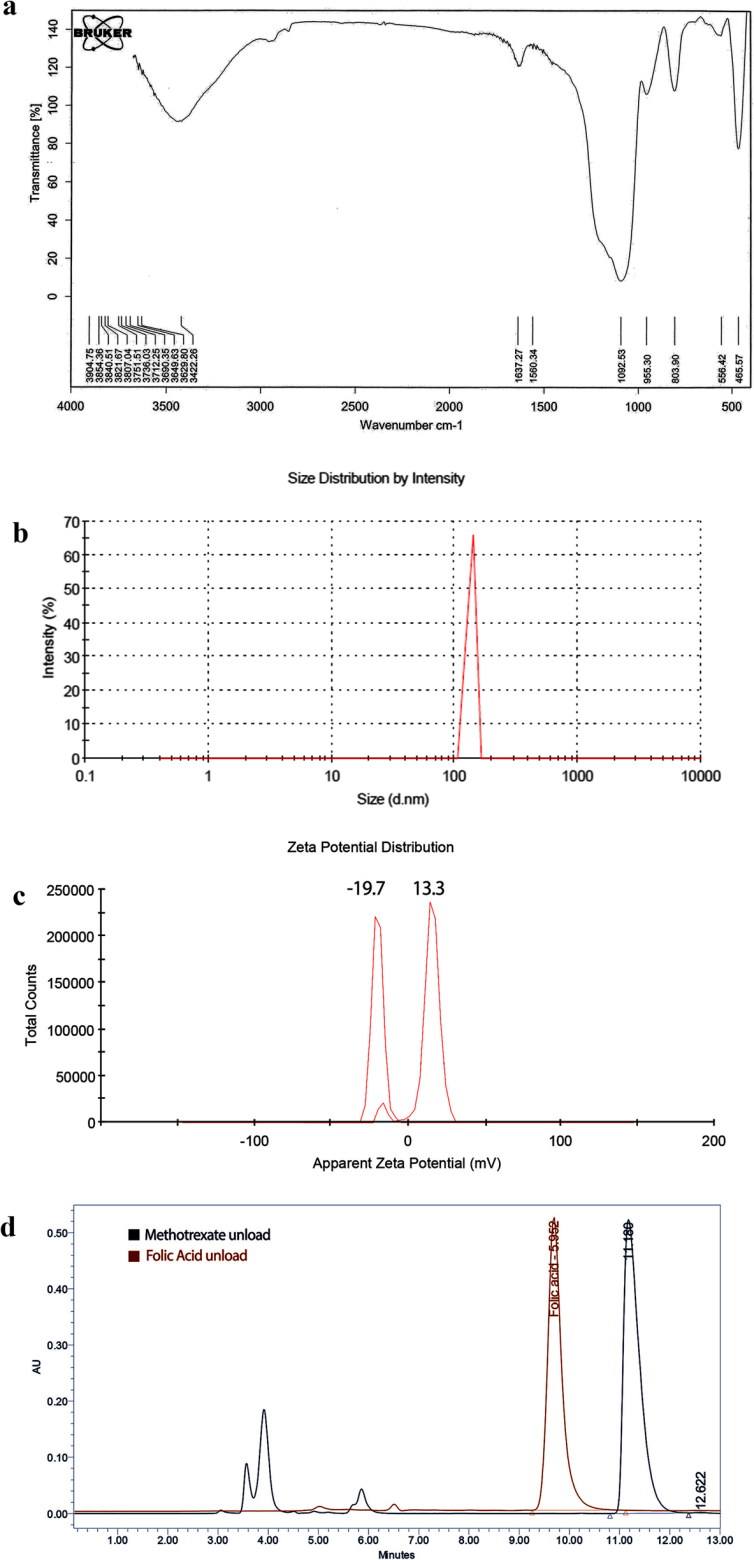
**a**) FTIR spectra of Au@SiO_2_ nanoparticles, **b**) size distribution of FA-MTX conjugated Au@SiO_2_ NPs measured by dynamic light scattering (DLS) **c**) The zeta potential of Au@SiO_2_ and FA-MTX conjugated Au@SiO_2_ NPs measured by dynamic light scattering (DLS) at pH=7.4 and T=25 °C, **d**) Chromatogram of unloaded MTX and FA separated from FA-MTX conjugated Au@SiO_2_ NP measured simultaneously by HPLC method

The Au@SiO_2_ NPs was produced in four steps: 1-synthesis of SiO_2_ nanoparticles, 2-addition of thiol containing linker to SiO2 NPs, 3-synthesis of gold nanoparticles, and 4-attaching the gold nanoparticles to the surface of SiO2-linker complexes (Fig. 1). The successful synthesis of Au@SiO_2_ was confirmed by FTIR (Fig. 2a). The Si–O–Si peak was appeared around 1088 cm^-1^. A wide peak at 3000–3700 and 803 cm^–1^ is credited to the stretching and out-of plane bending of free-silanol O–H groups, respectively. The aliphatic C–H stretching vibration was showed as strong peak at 2950 cm^–1^. The C–O of three methoxy silane groups was showed by peak at 1191 cm^–1^.

Dynamic light scattering (DLS) measurements indicated that FA-MTX conjugated Au@SiO_2_ NPs size was in the nanometer range (105±2.3 nm) with narrow size distribution (Fig. 2b).

Zeta potential is an important physicochemical parameter that influences the stability of nanosuspensions. Extremely positive or negative zeta potential values cause larger repulsive forces. On the other hand, the high charge of the particles, whether positive or negative, causes the NPs to be absorbed by the phagocytes of the liver and disposed of the body. In the case of a combined electrostatic and steric stabilization, a minimum zeta potential of ± 20 mV is desirable [69–71]. The zeta potential data of NPs were compared before and after loading with the MTX-FA at pH=7.4 and T=25 °C (Fig. 2c). The obtained zeta potentials of Au@SiO_2_ NPs were +13.3 mV which after dual drug loading decreased to -19.7 mV which were in the desirable range. The MTX and FA had a negative net charge at pH (7.4) above its pka (3.8 and 4.8, 3.5 and 4.3), due to the de-protonation of two carboxylic acid groups in its structure ([72], https://pubchem.ncbi.nlm.nih.gov.). Therefore, after MTX and FA simultaneous loading on Au@SiO_2_ NPs, the net charge became negative.

**Fig. 3:**
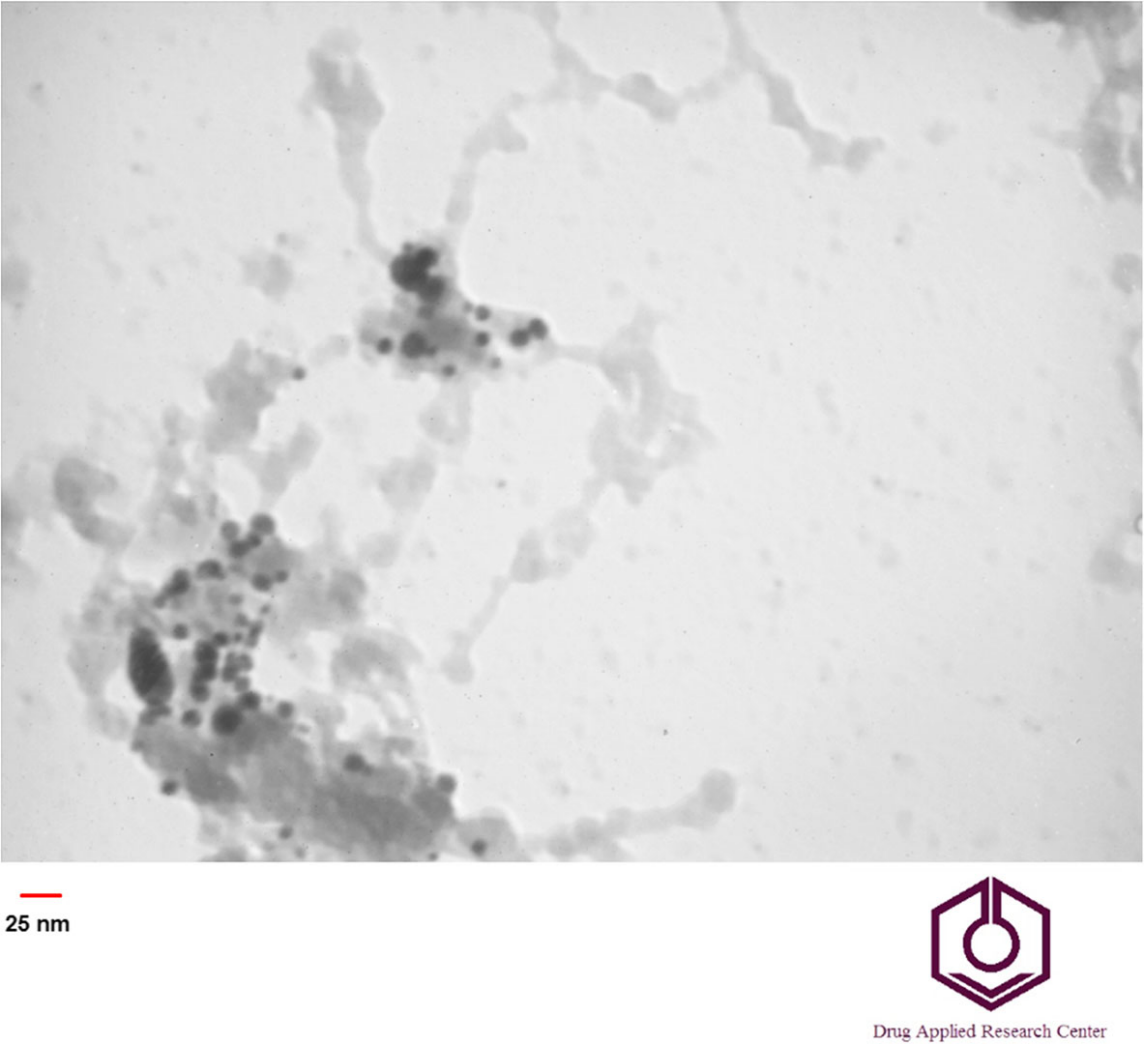
TEM image of Au@SiO_2_ nanoparticles

TEM analysis provides the definite individual particle size. Au nanoparticle was seen as dark spheres dispersed on the SiO2 NPs as a gray bed layer. TEM images confirmed that the Au@SiO2 NPs have synthesized with homogenous spherical shape in which the particles average size was around 25nm (Fig. 3).

### Drug loading

Herein, folate is mediating the increased uptake of GNPs in the certain types of cancer cells that overexpressed folate receptor through receptor mediated endocytosis to overwhelm the low efficacy of internalization of GNPs, therefor the folate receptor known as a tumor marker and folate use increasingly for tumor targeting [67, 73].

After MTX and FA molecules conjugation into Au@SiO_2_ NPs, the zeta potential changed from +13.3 to -19.7 mV. The estimated pKa values of the two carboxylic acid moieties of MTX is 3.8, 4.8 and FA is 3.5 and 4.3 [72], https://pubchem.ncbi.nlm.nih.gov. Therefore, due to de-protonation of two carboxylic acid groups of MTX and FA at pH 7.4 which is above their pka, the net charge became negative and indicating the successful conjugation of MTX and FA on Au@SiO_2_ NPs. De Ying Tian et al shows that MTX loading on Au nanoparticles by 18 and 30 mm diameter are 15 ± 0.4% and 10 ±1.0% respectively [74]. In this study FA and MTX were loaded in Au@SiO2 NPs with encapsulation efficiency of 22.6 and 77.5%, respectively. Chromatogram of simultaneous MTX and FA assessment peak has shown in Fig. 2d.

### Cell uptake

**Fig. 4:**
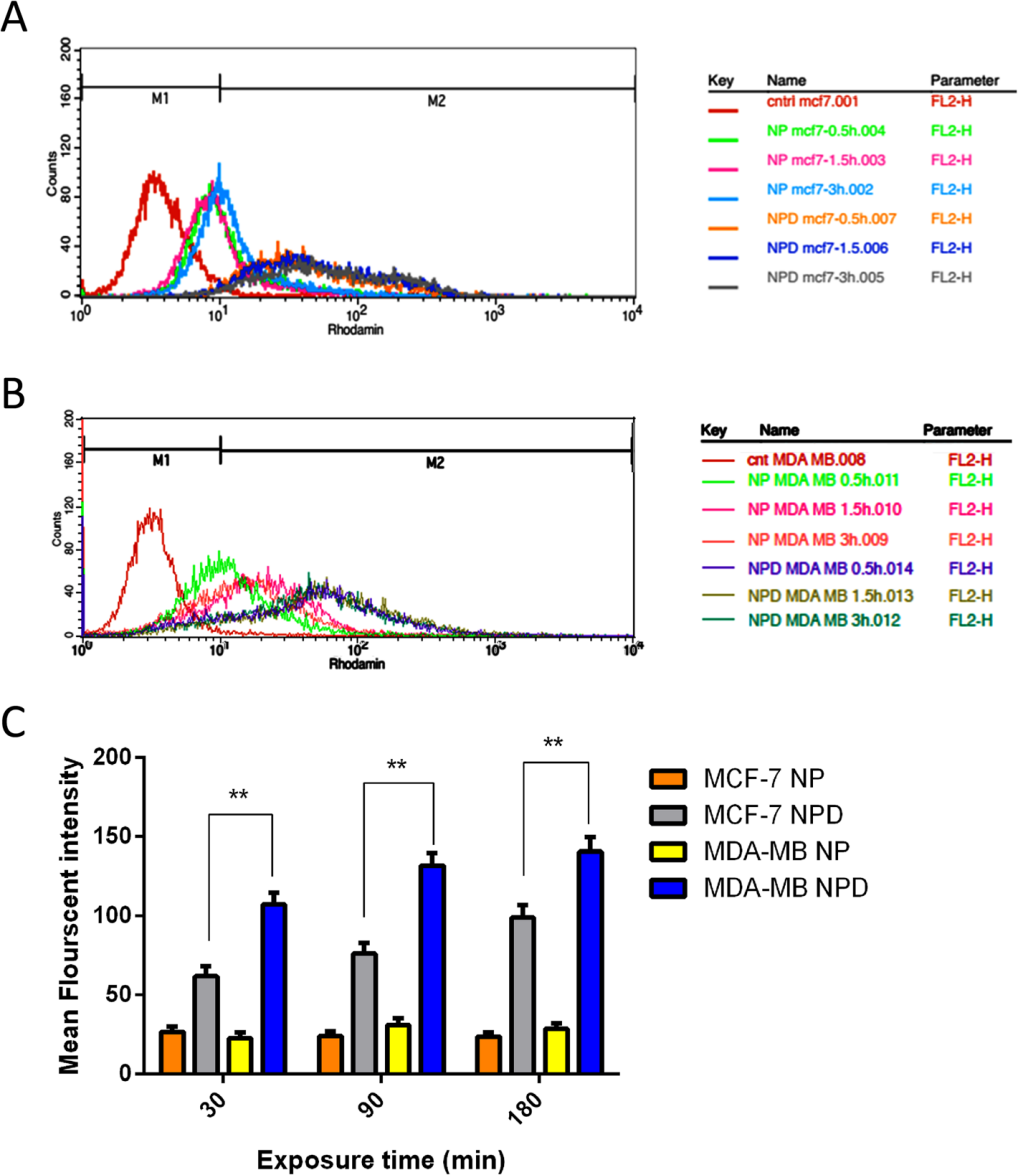
Quantitative cell uptake assay of rhodamine B-labelled Au@SiO_2_ nanoparticles (NP) or rhodamine B-labelled MTX-FA loaded Au@SiO_2_ nanoparticles (NPD) in MCF-7 (**a**) and MDA-MB-231(**b**) cell lines for exposure durations of 0.5 h, 1.5 h and 3h obtained by flow cytometry. Non-treated cells of both cell lines were employed as negative control. **c** Comparison of mean fluorescent intensity of rhodamine B-labelled Au@SiO_2_ nanoparticles (NP) or rhodamine B-labelled MTX-FA loaded Au@SiO_2_ nanoparticles (NPD) for exposure durations of 0.5 h, 1.5 h and 3h obtained by flow cytometry

**Fig. 5:**
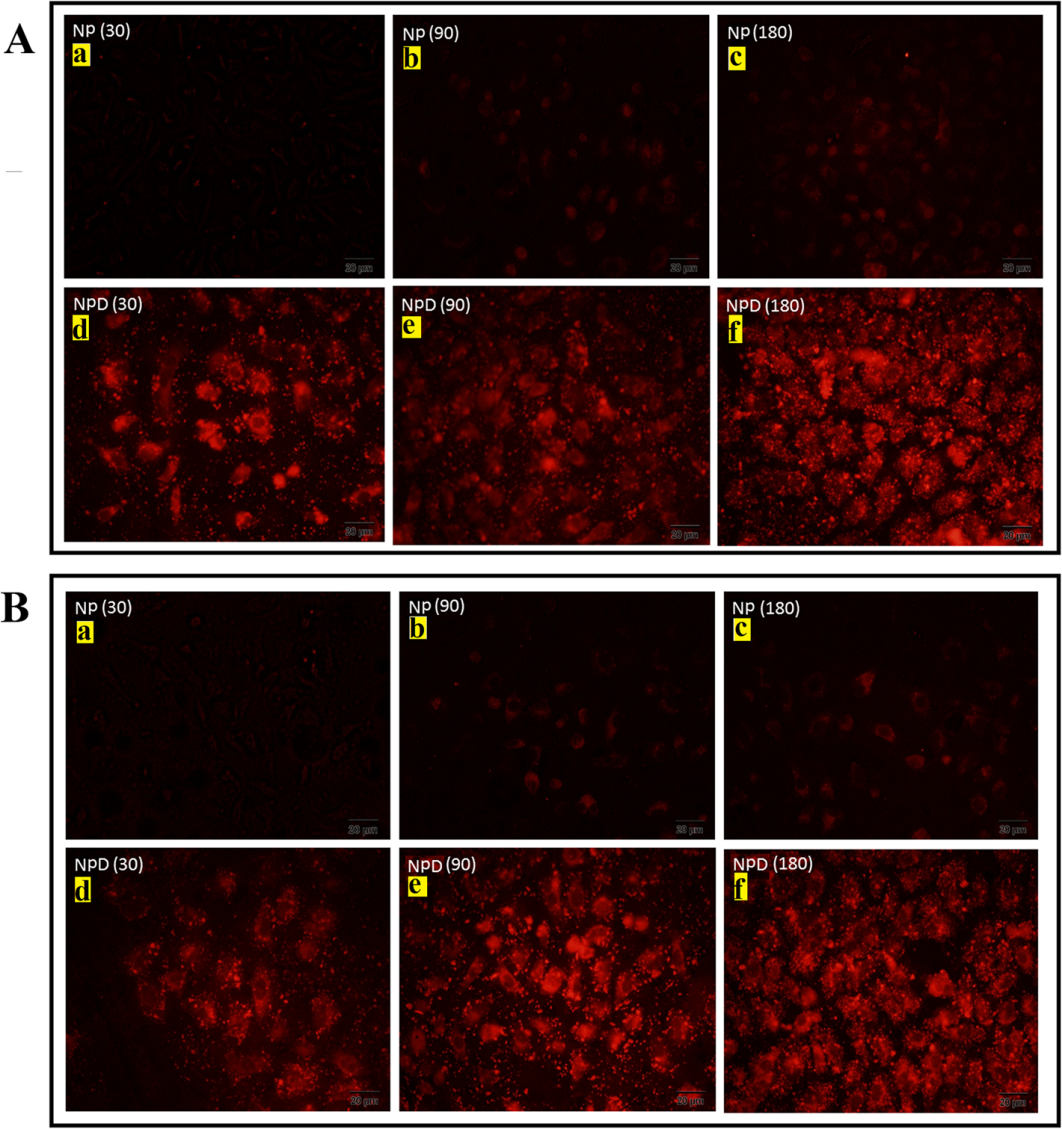
**A** Qualitative cell uptake assay using Rhodamine B-labelled Au@SiO_2_ nanoparticles (NP) in MCF7 with exposure durations of 30 (**a**), 90 (**b**) and 180 (**c**) min or rhodamine B-labelled MTX-FA loaded Au@SiO_2_ nanoparticles (NPD) with exposure durations of 30 (**d**), 90 (**e**) and 180 (**f**) min and (**B**) Qualitative cell uptake assay using Rhodamine B-labelled Au@SiO_2_ nanoparticles (NP) in MDA-MB-231 with exposure durations of 30 (**a**), 90 (**b**) and 180 (**c**) min or rhodamine B-labelled MTX-FA loaded Au@SiO_2_ nanoparticles (NPD) with exposure durations of 30 (**d**), 90 (**e**) and 180 (**f**) min captured by florescent microscopy

Because the intracellular photothermal agents can improve the efficiency of photothermal cancer therapy [75], it has been believed that cell internalization of photothermal materials is necessary. *In vitro* cellular uptake test was performed using human breast cancer MDA-MB-231 cells, known to highly overexpress the folate receptor [40]. To study the role of FA as a targeting agent and the efficiency of the surface coating on the uptake of the Au@SiO_2_ NPs by target cells, MCF-7 and MDA-MB-231 cells were treated with Au@SiO2 NPs and MTX-FA loaded Au@SiO2 NPs. The mean florescent intensity results of cell uptake were shown in Fig. 4. The results showed that Au@SiO2 NPs uptake in both MCF-7 and MDA-MB-231 was increased over cell culture time for all samples (Figs. 4 and 5). Also, after surface decoration of Au@SiO2 NPs with MTX and FA, the cells uptake was increased significantly on both MCF-7 and MDA-MB-231 as folate receptor expressing cells. MTX-FA-loaded Au@SiO2 NPs uptake into MDA-MB-231 cells was greater than MCF-7. Because MDA-MB-231 cells express higher levels of surface folate receptors so high portion of folate receptor targeted NPs were entered via receptor mediated endocytosis mechanism resulting in higher cellular uptake. In another study, the increased cell internalization of the folate conjugated NPs is occurring just in the cancer cells that overexpress the aHFR and not in the healthy cells that has less cell surface express of aHFRs [40]. Conjugation of Au@SiO2 NPs with FA can facilitate the cell uptake of NPs and methotrexate, leading to boosted toxicity toward to MDA-MB-231 cells [76].

### Cytotoxicity assay

**Fig. 6:**
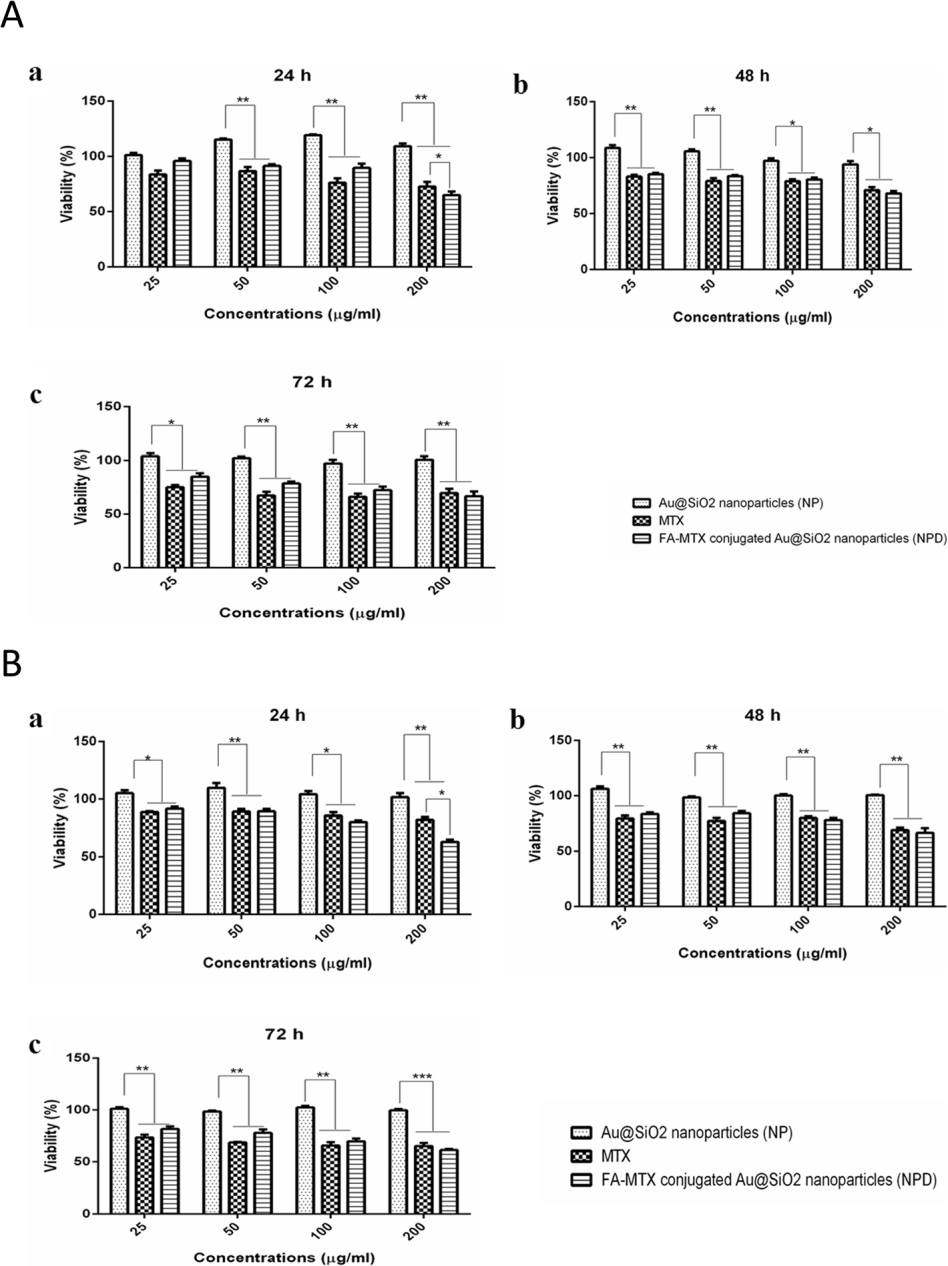
**a** MCF-7 and (**b**) MDA-MB-231 cells growth inhibition rates after treatment with different concentration of NP, MTX and FA-MTX loaded Au@SiO_2_ nanoparticles (NPD) after exposure time of 24, 48 and 72h

In vitro cellular cytotoxicity studies of the free MTX, blank Au@SiO_2_ NPs and MTX-FA conjugated Au@SiO_2_ NPs, were evaluated by MTT assay for 24, 48, and 72 h (Fig. 6). The MTT assay results showed that Au@SiO_2_ NPs had no cytotoxic effect on the MCF-7 and MDA-MB-231 cell lines. Furthermore, to compare the cytotoxicity effects of both free MTX and MTX-FA conjugated Au@SiO_2_ NPs, the same concentration of MTX (25, 50, 100 and 200μg/mL) was used for all treatment times. Cell cytotoxicity results indicate that free MTX or MTX-FA conjugated Au@SiO_2_ NPs showed around 10-25% mortality rate in both cell lines after 24h of treatment. Previous studies reported the proliferative effect of gold nanoparticles on different cell lines like murine osteoblast MC3T3-E1 cells and human periodontal ligament stem cells under in vitro conditions. Our results are in line with these studies and Figure 6a and b shown the proliferative effect of free Au@SiO2 NPs. Therefore the equal cytotoxic effect of MTX and FA-MTX loaded Au@SiO2 nanoparticles (NPD) may be due to this phenomena [77, 78].

### Laser irradiation

**Fig. 7:**
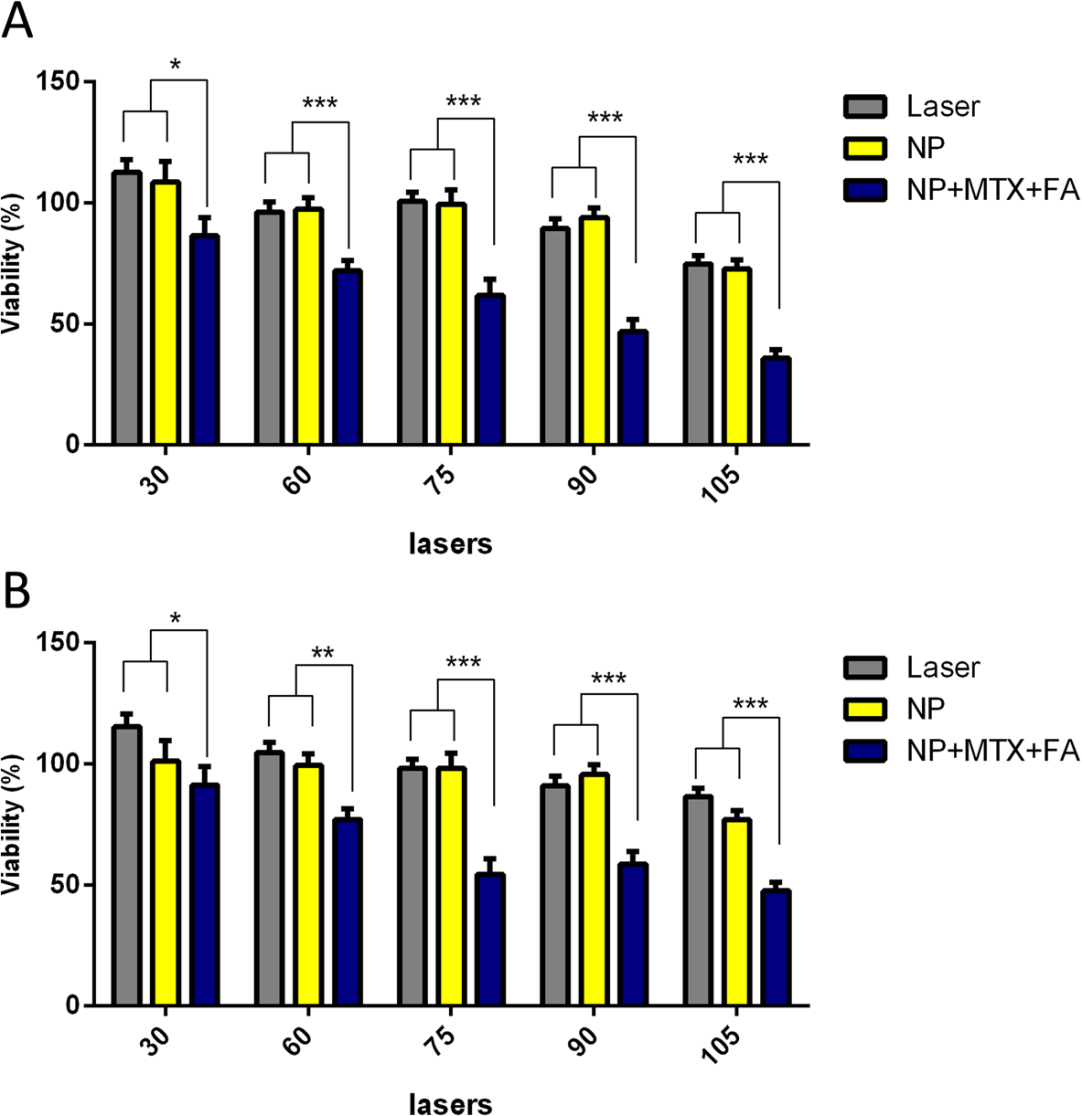
A comparison of cell growth inhibition rates exposed to different laser powers (30, 60, 75, 90 and 105 J/cm^2^) for treatment groups of laser alone, laser + Au@SiO2 nanoparticles and laser + MTX-FA loaded Au@SiO2 nanoparticles directed for two cell line MCF-7 (**a**) and MDA-MB-231(**b**) with subsequent checking after 24h

In this study, the cell viability of MCF-7 and MDA-MB 231 cells treated with Au@SiO_2_ NPs and MTX-FA loaded Au@SiO_2_NPs after laser irradiation with dose in the range of 30-105 J/cm^2^ was investigated by MTT assay. The mortality rate of MCF-7 and MDA-MB-231 cells treated with MTX- FA loaded Au@SiO_2_ NPs (the MTX concentration was 100 μg/mL) after LLLT at a dose of 75 J/cm^2^ were about 39 and 45.5%, respectively. While at the same condition the cells treated with Au@SiO_2_ NPs after laser irradiation or laser alone did not show obvious cell death. Also by increasing the laser dose to 105 J/cm^2^ the mortality rate of both cell lines increased to 60-75%, while both cell liness treated with Au@SiO_2_ NPs +laser or laser alone at the same irradiation dose showed no cytotoxic effect. The IC50 value for MCF-7 and MDA-MB-231 cells after combination therapy with MTX-FA loaded Au@SiO_2_ NPs (MTX dose of 100 μg/mL) and LLLT were obtained at a dose of 90 and 75 J/cm^2^, respectively. On the other hand the mortality rate of MTX and MTX-FA loaded Au@SiO_2_NPs without laser irradiation at MTX dose of 100 μg/mL (selected dose for laser therapy study) was between 15-25% in both cell lines. These results indicated that the combination of MTX-FA loaded Au@SiO_2_NPs and laser therapy showed a synergistic effect in both cell lines and significantly decreased the cell viability (*p*<0.001) compared to cells received only laser irradiation. These results indicated that NPs treatment, especially with targeting strategy can improved the efficacy of laser therapy in breast cancer cell destruction (Fig. 7).

### Apoptosis study by DAPI

**Fig. 8:**
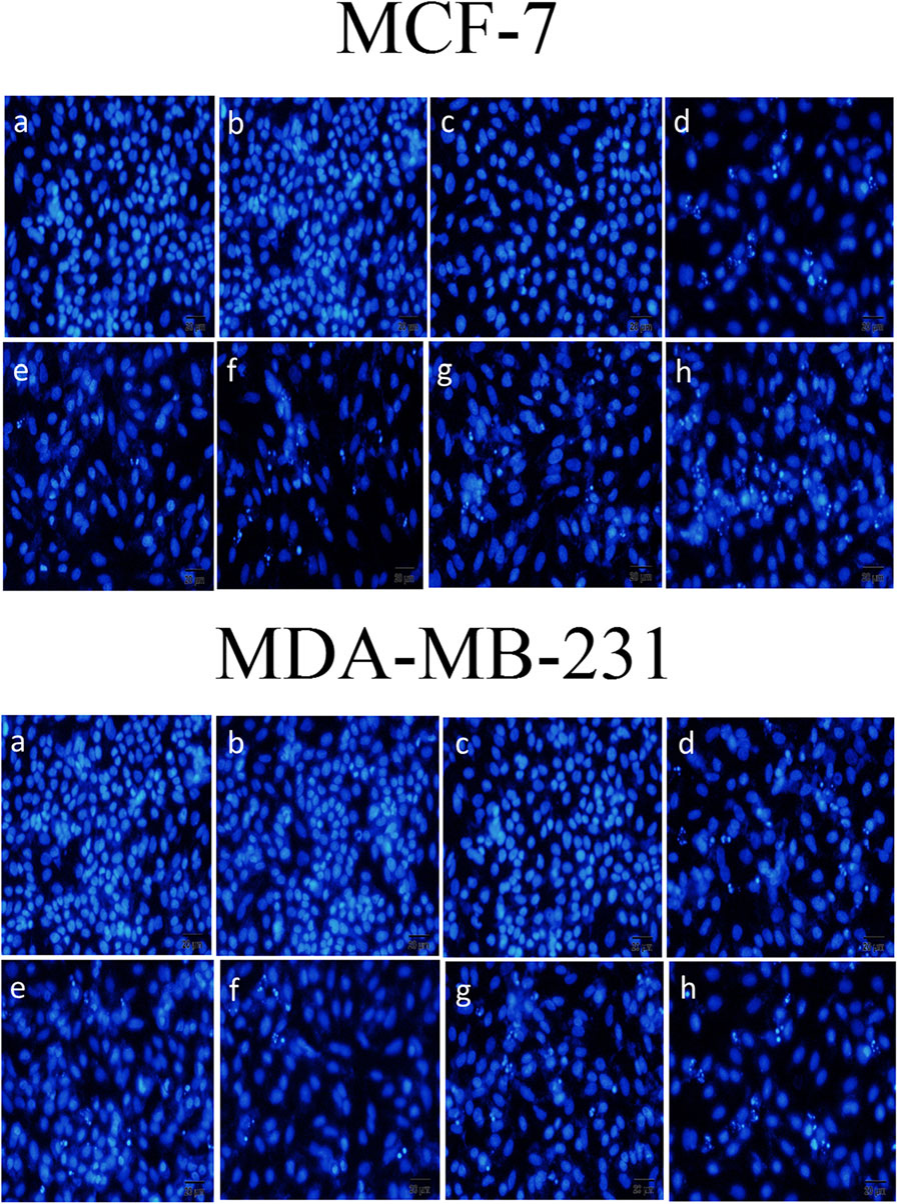
Apoptosis assay using DAPI staining for MCF-7 or MDA-MB-231 cells, images captured using an inverted microscope. The untreated cells as the negative control (**a**), cells treated with laser (75 J/cm^2^) as positive control (**b**) cells treated with Au@SiO_2_ nanoparticles (NP (**c**), cells treated with laser (75 J/cm^2^) and Au@SiO_2_ nanoparticles (NP) (**d**), cells treated with MTX without laser irradiation (**e**), cells treated with MTX and laser irradiation (**f**), cells treated with MTX-FA-loaded Au@SiO_2_ nanoparticles (NPD) without laser exposure (**g**), and cells treated with MTX-FA-loaded Au@SiO_2_ nanoparticles (NPD) with Laser exposure (**h**)

The apoptosis were studied in MCF-7 and MDA-MB-231 cells after treatment with Au@ SiO_2_ NPs; MTX-FA loaded Au@ SiO_2_ NPs with or without laser to know if laser treatment could enhance the efficacy of chemotherapy. Our results summarized in Fig. 8 indicated that normal MCF-7 and MDA-MB-231 cells without any treatment set as control as well as cells treated with laser alone or free Au@SiO_2_ NPs without laser irradiation had typical nuclei, lacking any apoptosis. However, MCF-7 and MDA-MB-231 cells treated with free MTX, Au@ SiO_2_ NPs with laser irradiation and MTX and FA loaded Au@SiO_2_ NPs without laser irradiation showed partial apoptotic nuclei (Fig. 8). The cells treated with MTX and FA loaded Au@SiO_2_ NPs in combination with laser irradiation (810 nm, 75 J/cm^2^, 139 sec) showed a major drop in MCF-7 and MDA-MB-231 cell population. Therefore, laser irradiation efficacy was enhanced after MTX/FA loaded Au@SiO_2_ NPs uptake on MCF-7 and MDA-MB-231 cells. Hence, the novel developed MTX and FA loaded Au@SiO_2_ NPs has the capability of augmenting the photothermal effects by highly fragmented cell nuclei, a radical rise in cell loss and complete damage of cells.

### Cell cycle

**Fig. 9:**
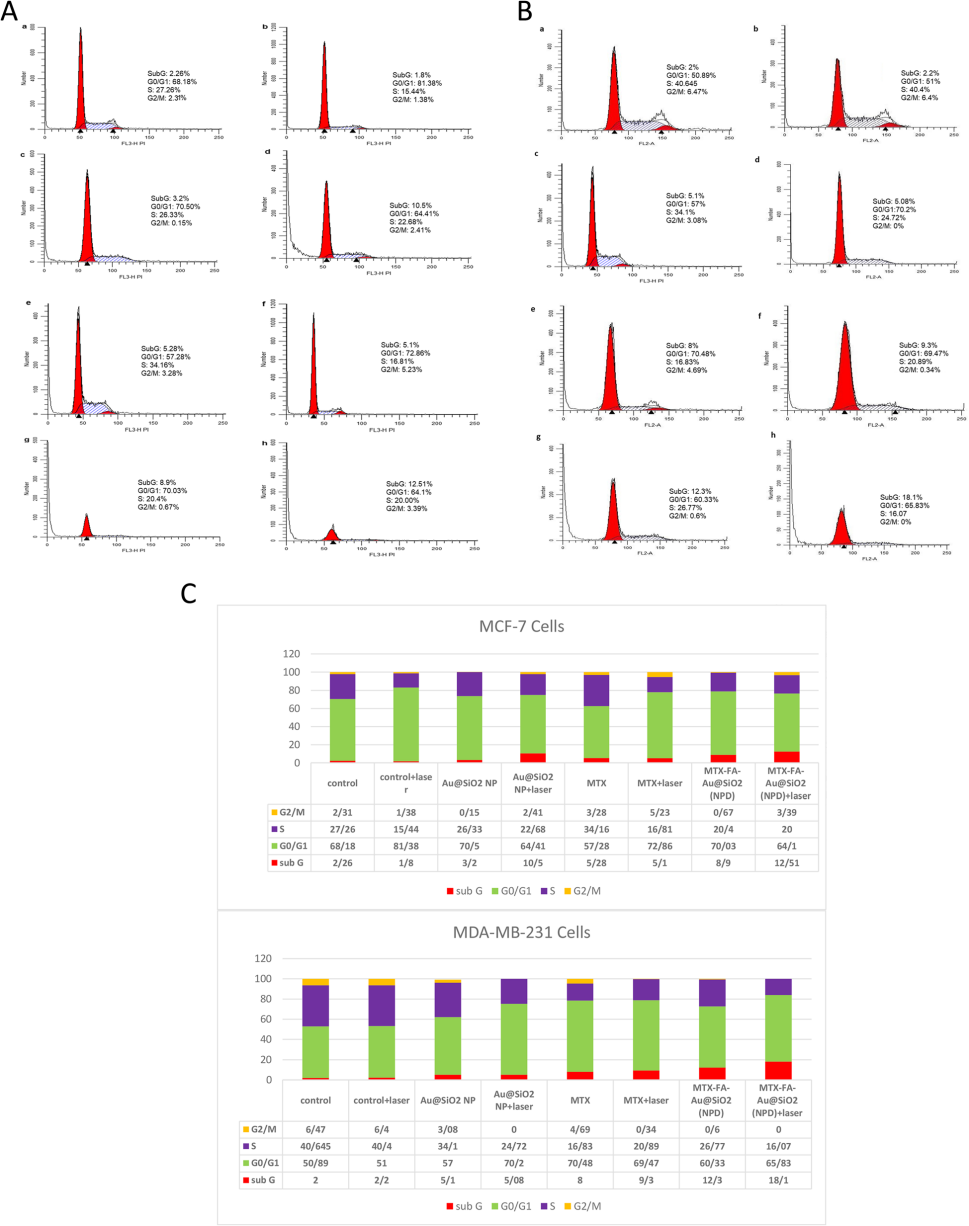
Cell cycle distributions investigated for MCF-7 (**A**) or MDA-MB-231 (**B**) cells. The untreated cells as negative control (**a**), cells treated with laser (75 J/cm^2^) as positive control (**b**) cells treated with Au@SiO_2_ nanoparticles (NP) (**c**), cells treated with laser (75 J/cm^2^) and Au@SiO_2_ nanoparticles (NP) (**d**), cells treated with MTX without laser irradiation (**e**), cells treated with MTX and laser irradiation (**f**), cells treated with MTX-FA-loaded Au@SiO_2_ nanoparticles (NPD) without laser exposure (**g**), and cells treated with MTX-FA-loaded Au@SiO_2_ nanoparticles (NPD) with Laser exposure (**h**), **C**) Quantitative results of cell cycle arrest and its distribution

Cell cycle distributions after treatment with MTX, Au@SiO_2_ NPs and MTX and FA loaded Au@SiO_2_ NPs either in combination with LLLT (75 J/cm^2^) or without laser irradiation was studied in both MCF-7 and MDA-MB-231 cells using flowcytometry and PI staining of DNA. Our study indicated that in MDA-MB-231 or MCF-7 cells, the percentage of non-treated cells (control group) were actively in phase S (Fig. 9a, b). Using 75 J/cm^2^ laser treatments reduced the percentage of cells in S-phase in a non-significant manner. On the other hand the cells irradiated with LLLT without NP and drug treatment showed the significant increase in Go/G1 cell population indicated the safety of LLLT alone. Also NPs treatment did not disturb the cell cycle in both cell lines. Treatment of cells with free MTX in the absence or presence of laser irradiation showed some disturbances in cell cycles, including reduction of cells in S-phase. Using NPD alone reduced the cells in S-phase. And interestingly using MTX and FA loaded Au@SiO_2_ NPs (NPD) enhanced the cell percentage in sub Go/G1 as a sign of apoptosis [72]. Also the percentage of the MDA-MB-231 cells present in sub Go/G1 (around 18%) were significantly higher than MCF-7 cells (12%) in MTX and FA loaded Au@SiO_2_ NPs (NPD) treatment group due to the higher uptake of NPs in MDA-MB-231 cells.

Ramos *et al*, showed that in tumor cells, LLLT increases the percentage of cells in S and G2 /M phases, also they detected a reduction in proliferation and enhancing in senescence [79]. The cell cycle study after LLLT (15 J/cm2) showed a G1 arrest, which is in line with growth stopover in irradiated TK6 cells [80]. Another group reported that PTT is primarily disturbing cells in the S phase and increasing the cell population and arrest in the G2/M phase [81]. As a result, PTT can induce radio-sensitization of the cells via disturbing cell cycle [82]. Their results are in accordance with our study, which showed cell cycle disturbance and reduction of cells in S-phase. Our study also showed the increase in population of apoptotic cells (sub Go/G1) after combination chemo-photothermal therapy. Therefore, applying a combination of LLLT and MTX and FA loaded Au@SiO_2_ NPs (NPD) as breast cancer targeted nanoparticles could enhance the breast cancer therapy efficacy.

## Conclusions

In this study MTX and FA loaded Au@SiO_2_ NPs was designed for target breast cancer therapy in combination with LLLT as noninvasive, FDA approved laser therapy. MTX and FA loaded Au@SiO_2_ NPs with spherical morphology and mean diameter of 25nm and surface charge of -19.7 was obtained. This size and surface charge is in a suitable range to increase the bio-distribution of NPs. The successful targeted strategy of this novel developed NPs was approved with a higher cellular uptake percentage of MDA-MB-231 compared to MCF-7 as two breast cancer cell lines with different folate receptor expression. The MTT assay, DAPI staining and cell cycle study's results indicated that the combination of chemo-photothermal therapy showed synergistic effect and the cytotoxicity and apoptosis effect on both breast cancer cell lines especially on MDA-MB-231 cells was increased significantly(*p*<0.001). Since the Au@SiO_2_ nanoparticles or LLLT showed no cytotoxic effects, it can be concluded that our therapeutic design has synergistic effects on targeted site. The findings of this study could be useful for designing future cancer therapy programs using bio-chemotherapy combined with low level lasers.

## Data Availability

Not applicable

## Abbreviations

Au@SiO_2_: Silica coated gold
BC: Breast cancer
DHFR: Dihydrofolate reductase
DMSO: Dimethyl Sulfoxide
FA: Folate
LLLT: Low level laser therapy
LSPR: Localized surface plasmon resonance
MSN: Mesoporous silica nanoparticle
MTX: Methotrexate
NIR: Near-infrared
NPs: Nanoparticles
PTT: Photothermal therapy
RhoD: rhodamine B
TFSNPs: Thiol-functionalized silica-coated nanoparticles
